# Political ideology, sociodemographic factors, and social media health information engagement among U.S. adults: evidence from HINTS 2024

**DOI:** 10.3389/fdgth.2026.1883403

**Published:** 2026-08-12

**Authors:** Jingjing Gao, Jason H. Windett

**Affiliations:** 1 Department of Management, Policy, and Community Health, University of Texas Health Science Center at Houston School of Public Health, Houston, TX, United States; 2 Public Policy Ph.D. Program, Department of Political Science and Public Administration, School of Data Science, University of North Carolina at Charlotte, Charlotte, NC, United States

**Keywords:** digital divide, digital health, digital public health, health communication, health information, HINTS, political ideology, public health

## Abstract

**Background:**

Social media platforms have become increasingly important sources of health-related information for the U.S. public. At the same time, political polarization and sociodemographic inequalities may be associated with how individuals engage with health information on digital platforms. Understanding the relationship between political ideology, socioeconomic factors, and social media health information engagement has important implications for digital public health communication and health equity.

**Objective:**

This study examined associations between political ideology, sociodemographic characteristics, and multiple dimensions of social media health information engagement among U.S. adults using nationally representative Health Information National Trends Survey (HINTS) 2024 data.

**Methods:**

Cross-sectional nationally representative data from HINTS 2024 were analyzed. Five dimensions of social media engagement were examined, including social media visitation, sharing personal content, sharing general content, interacting with others online, and watching videos on social media platforms. An overall social media engagement index was constructed using the row mean of these measures. Multivariable linear regression analyses were conducted to evaluate associations between political ideology, demographic and socioeconomic characteristics, and social media engagement behaviors.

**Results:**

Age was consistently and negatively associated with all dimensions of social media engagement, including social media visitation (*β* = −0.025, *p* < 0.001), content sharing, online interaction, video consumption, and overall engagement. Female participants generally demonstrated higher levels of social media engagement than male participants. Hispanic participants reported significantly greater video-based engagement (*β* = 0.301, *p* < 0.001) and higher overall social media engagement (*β* = 0.090, *p* < 0.001), while non-Hispanic Asian participants demonstrated significantly greater engagement across several behaviors, including content sharing and video consumption. Higher educational attainment was consistently associated with greater overall social media engagement, particularly among individuals with bachelor's and post-baccalaureate education. Political ideology demonstrated more limited but still notable associations with engagement behaviors. Compared with very liberal participants, somewhat conservative individuals reported greater social media visitation frequency (*β* = 0.243, *p* < 0.05), while very conservative individuals demonstrated higher levels of online interaction (*β* = 0.159, *p* < 0.01) and overall social media engagement (*β* = 0.078, *p* < 0.05).

**Conclusion:**

Sociodemographic factors, particularly age, education, and race and ethnicity, were strongly associated with social media health information engagement among U.S. adults. Political ideology demonstrated smaller but meaningful associations with selected digital engagement behaviors. These findings highlight important digital public health disparities in how individuals engage with health-related content on social media platforms.

## Introduction

Social media platforms have become increasingly important sources of health-related information for the U.S. public. Individuals increasingly rely on social media platforms to obtain health information, share personal experiences, access educational resources, communicate with online communities, and engage with healthcare-related content. Digital communication environments now play a major role in shaping public understanding of health, disease prevention, healthcare utilization, and public health messaging (1–6). The rapid growth of social media use has transformed how health information is disseminated, consumed, and discussed across diverse population groups.

Although social media platforms may improve access to health information and facilitate communication, important disparities exist in how individuals engage with digital health information environments. Prior research suggests that social media engagement behaviors differ substantially across demographic and socioeconomic groups. Age, educational attainment, household income, race and ethnicity, and digital literacy may influence both the frequency and type of social media engagement behaviors (7–12). These disparities may contribute to unequal access to health information, online social support, and digital health resources, potentially reinforcing existing public health inequalities. Prior studies using the Health Information National Trends Survey have documented substantial variation in digital health and social media engagement across population groups. Earlier HINTS analyses found that age, sex, educational attainment, income, and race and ethnicity were associated with the use of online health-information tools and social media for health purposes (13–18). For example, nationally representative HINTS 2019 findings showed that younger adults and women were more likely to engage in several social media health activities, including visiting social networking sites, sharing health information, participating in online support groups, and watching health-related videos (15). Other HINTS-based research has similarly identified persistent differences in eHealth use and health-information seeking by socioeconomic and demographic characteristics (19). These findings suggest that digital engagement involves not only access to technology but also differences in how individuals seek, exchange, and use health information online.

Political ideology may also be associated with social media health-information engagement through differences in institutional trust, media preferences, information networks, and responses to public health communication. Research conducted during the COVID-19 pandemic demonstrated that health communication became closely connected with political identity and partisan conflict (20–25). Social media environments may intensify these differences by facilitating selective exposure, ideologically homogeneous networks, and the rapid circulation of emotionally salient or inaccurate information (26, 27). However, the effects of social media are not uniform, and exposure to opposing political viewpoints does not necessarily reduce polarization and may, under some circumstances, reinforce existing political attitudes. Accordingly, political polarization provides an important theoretical context for examining health communication, although self-reported ideology alone does not directly measure ideological, affective, or false polarization.

Political ideology is generally understood as an organized system of beliefs, values, and political orientations through which individuals interpret social arrangements, public institutions, and policy choices. Converse described ideological belief systems in terms of the degree to which political attitudes are connected or constrained by broader organizing principles (28). In contemporary U.S. political research, ideology is frequently represented through self-placement along a liberal-conservative continuum (29). Liberal political thought has traditionally emphasized individual liberty, justification of political authority, and protection of personal rights, while conservative traditions have generally emphasized continuity, established institutions, social order, and caution toward rapid political change (30–35). However, neither tradition is conceptually uniform, and individuals may hold different positions across social and economic policy domains. Therefore, the present study treats political ideology as self-identified orientation on the U.S. liberal-conservative spectrum rather than as membership in a single, fully coherent philosophical doctrine. Political polarization is distinct from political ideology and can take several forms. Ideological polarization refers to increasing divergence in political values, policy preferences, or ideological self-placement, often reflected in movement away from moderate positions (36, 37). Affective polarization refers to growing dislike, distrust, or perceived threat toward members of opposing political groups (38, 39). Perceived or false polarization occurs when individuals overestimate the extent of disagreement or ideological distance between political groups. These dimensions require different measurement approaches and should not be inferred solely from respondents' self-placement on a liberal-conservative scale. The present study therefore examines political ideology as an individual characteristic and treats political polarization only as a contextual framework.

Social media are embedded within a broader political communication environment in which citizens, political actors, journalists, and organizations can produce, amplify, and respond to information. Rather than functioning only as passive distribution channels, social media may facilitate political awareness, discussion, participation, and direct interaction among users and political actors (40). Social media may also influence which issues receive attention by weakening some traditional gatekeeping processes and linking the agendas of political parties, individual politicians, news organizations, and the public (41). However, these agenda-setting relationships are often reciprocal, and social media operate alongside traditional journalism within a hybrid media system rather than replacing it (42). These features may contribute to differences in how ideological groups encounter, share, and discuss health-related information online.

Health misinformation is an additional concern within social media-based health communication. False information may spread rapidly online, while users may encounter difficulty distinguishing reliable evidence from misleading, incomplete, or ideologically framed claims (43, 44). National research indicates that a substantial proportion of social media users perceive high levels of health misinformation, and exposure to or perceptions of misinformation may be associated with lower trust in healthcare institutions (45–47). Because political ideology may be related to trust, information-source preferences, and interpretations of scientific evidence, examining ideological differences in social media health engagement may help clarify which forms of digital participation vary across political groups.

Despite an expanding literature on eHealth use, social media engagement, political communication, and health misinformation, several gaps remain. Prior HINTS-based studies have primarily examined sociodemographic predictors of general eHealth use or selected social media activities, while less nationally representative research has evaluated political ideology alongside multiple forms of health-related social media engagement. In addition, visiting social media, sharing personal information, sharing general health information, interacting with peers, and watching videos represent conceptually distinct forms of engagement that may have different correlates. The present study addresses these gaps by examining political ideology and sociodemographic characteristics across five specific engagement behaviors and a validated overall engagement index using nationally representative HINTS 2024 data.

Therefore, this study used nationally representative Health Information National Trends Survey (HINTS) 2024 data to examine associations between political ideology, sociodemographic characteristics, and multiple dimensions of social media health information engagement among U.S. adults. Specifically, this study aimed to: (1) examine demographic and socioeconomic differences in social media health information engagement; (2) evaluate associations between political ideology and digital engagement behaviors; and (3) assess multiple dimensions of social media engagement, including social media visitation, content sharing, online interaction, video consumption, and overall engagement.

## Methods

### Data source and study population

This study utilized cross-sectional nationally representative data from the 2024 Health Information National Trends Survey (HINTS) administered by the National Cancer Institute. HINTS is designed to assess health communication, social media use, digital health behaviors, and health information access among U.S. adults. The 2024 cycle was selected because it included measures assessing political ideology alongside detailed social media health information engagement behaviors.

### Measures

#### Outcome variable

The primary outcome—social media health information engagement was assessed using five HINTS 2025 measures examining health-related social media behaviors during the previous 12 months, including visiting social media sites (SocMed_Visited), sharing personal health information (SocMed_SharedPers), sharing general health-related information such as news articles (SocMed_SharedGen), interacting with individuals who had similar health or medical issues online (SocMed_Interacted), and watching health-related videos on social media platforms (SocMed_WatchedVid). Response categories ranged from “Never” to “Almost every day” and were coded from 1 to 5, with 1 representing “Never” and 5 representing “Almost every day.” Higher values therefore indicated more frequent engagement. Responses classified as missing data, web partial responses, or multiple responses selected in error were recoded as missing before analysis. An overall social media health information engagement index (socmed_total) was constructed using the row mean of the five engagement measures, with higher scores indicating greater overall engagement with health-related social media activities. The overall engagement index was calculated among respondents with at least three valid responses across the five engagement items. The HINTS items assessed the frequency and type of health-related social media engagement but did not identify the specific social media platforms used. Consequently, platform-specific use and comparisons across Facebook, YouTube, Instagram, TikTok, X, or other platforms could not be examined.

#### Primary independent variable

Political ideology was assessed using self-reported political orientation measures available in HINTS. Respondents self-identified as very conservative, conservative, somewhat conservative, moderate, somewhat liberal, liberal, or very liberal. The measure represents self-placement on the contemporary U.S. liberal-conservative continuum rather than a multidimensional assessment of social and economic ideology. Political ideology was modeled categorically, with moderate respondents serving as the reference group. Responses classified as missing, not ascertained, web partial, multiple responses selected in error, or not applicable were treated as missing. This measure assesses ideological self-identification but does not directly measure ideological polarization, affective polarization, issue-based polarization, or perceived or false polarization.

#### Covariates

Covariates included age, sex, race and ethnicity, educational attainment, and household income. Age was treated as a continuous variable measured in years. Sex was coded as male or female. Race and ethnicity were categorized as non-Hispanic White, non-Hispanic Black, Hispanic, non-Hispanic Asian, and non-Hispanic Other. Educational attainment was classified into five categories: less than high school, high school graduate, some college, bachelor's degree, and post-baccalaureate education. Household income was categorized into six ordinal income groups ranging from the lowest income category to the highest income category, with higher values representing greater household income levels. All categorical variables were included in regression analyses using indicator coding.

The original HINTS 2024 public-use dataset included 7,279 respondents. Responses categorized as missing data, not ascertained, web partial, multiple responses selected in error, or blank were treated as missing. No missing values were imputed. Each regression model used complete-case analysis for the relevant engagement outcome, political ideology, age, sex, race and ethnicity, educational attainment, and household income. Because missingness varied across outcomes, the final analytic sample differed by model. The final unweighted sample sizes were 5,850 for social media visitation, 5,831 for sharing personal health information, 5,824 for sharing general health information, 5,843 for interacting with others online, 5,850 for watching health-related videos, and 5,849 for the overall engagement index.

### Statistical analysis

Survey-weighted descriptive statistics were calculated to characterize the study population and social media health-information engagement. Because the five individual engagement measures were ordinal frequency outcomes, associations with political ideology and sociodemographic characteristics were estimated using survey-weighted ordinal logistic regression models. Results are reported as adjusted odds ratios with 95% confidence intervals. The internal consistency and dimensionality of the overall engagement index were evaluated using inter-item correlations, standardized Cronbach's alpha, item-rest correlations, and exploratory factor analysis. The index was calculated as the mean of the five engagement items among respondents with at least three valid responses and was analyzed as a secondary continuous outcome using survey-weighted linear regression. All regression models adjusted for demographic and socioeconomic covariates, including age, sex, race and ethnicity, educational attainment, and household income. Taylor series linearization was used to account for the HINTS sampling weights, strata, and primary sampling units. Design-adjusted Wald tests were used to evaluate the joint association between political ideology and each outcome, and overall model significance was assessed using design-adjusted *F* tests. Statistical significance was defined as a two-sided *p* value <0.05.

## Results

### Descriptive characteristics

The original HINTS 2024 dataset included 7,279 respondents. Following outcome-specific complete-case analysis, the final regression samples ranged from 5,824 to 5,850 respondents. Model-specific sample sizes are reported in Table 2. Participants represented diverse demographic, socioeconomic, and political backgrounds. The majority of participants reported regular internet use and exposure to online health information sources.

Table 1 presents the descriptive characteristics of the study population and levels of social media health information engagement among U.S. adults in HINTS 2024. The analytic sample included 6,664 participants with a survey-weighted mean age of 58.03 years (SE = 0.65). The sample was relatively balanced by sex, including 4,017 female participants and 2,645 male participants, representing weighted proportions of 48.8% and 51.2%, respectively.

**Table 1:** Descriptive characteristics of the study population and social media health information engagement among U.S. adults, HINTS 2024.

Variable	*n*	Weighted %/mean ± SE
Age (years)	6,664	58.03 ± 0.65
Sex		
Male	2,645	51.2%
Female	4,017	48.8%
Race and ethnicity		
Non-Hispanic white	3,547	60.6%
Non-Hispanic black	968	11.1%
Hispanic	1,323	17.5%
Non-Hispanic Asian	342	5.6%
Non-Hispanic other	257	5.3%
Educational attainment		
Less than high school	443	6.2%
High school graduate	1,121	21.4%
Some college	1,941	38.6%
Bachelor's degree	1,863	20.0%
Post-baccalaureate	1,326	13.9%
Household income		
Lowest income group	1,123	15.1%
Income group 2	802	11.6%
Income group 3	782	11.6%
Income group 4	1,030	15.6%
Income group 5	785	12.4%
Highest income group	1,856	33.8%
Political ideology		
Very conservative	475	7.4%
Conservative	919	13.7%
Somewhat conservative	688	10.9%
Moderate	2,288	37.6%
Somewhat liberal	657	10.5%
Liberal	932	13.5%
Very liberal	467	6.4%
Social media health information engagement measures		
Visited social media platforms	2,942	2.47 ± 0.05
Shared personal content	7,048	1.13 ± 0.02
Shared general content	7,036	1.21 ± 0.02
Interacted with others online	7,034	1.19 ± 0.02
Watched videos on social media	6,853	1.76 ± 0.04
Overall social media engagement index	7,155	1.55 ± 0.02

Values are survey-weighted percentages or means ± standard errors. Social media engagement measures ranged from 1 to 5, with 1 indicating “Never” and 5 indicating “Almost every day.” Higher scores indicate more frequent engagement. The overall engagement index was calculated using the row mean of the five engagement measures.

Most participants identified as non-Hispanic White (*n* = 3,547; 60.6%), followed by Hispanic (*n* = 1,323; 17.5%), non-Hispanic Black (*n* = 968; 11.1%), non-Hispanic Asian (*n* = 342; 5.6%), and non-Hispanic Other racial and ethnic groups (*n* = 257; 5.3%). Regarding educational attainment, the largest proportion of participants reported some college education (*n* = 1,941; 38.6%), followed by high school graduates (*n* = 1,121; 21.4%), bachelor's degree holders (*n* = 1,863; 20.0%), and post-baccalaureate degree holders (*n* = 1,326; 13.9%). Approximately one-third of participants were classified in the highest household income group (33.8%). Politically, moderate ideology represented the most common orientation (*n* = 2,288; 37.6%).

Across the five measured activities, social media visitation was reported most frequently (*n* = 2,942; mean = 2.47 ± 0.05), followed by watching videos on social media platforms (*n* = 6,853; mean = 1.76 ± 0.04). Lower engagement levels were observed for sharing personal content (*n* = 7,048; mean = 1.13 ± 0.02), sharing general content (*n* = 7,036; mean = 1.21 ± 0.02), and interacting with others online (*n* = 7,034; mean = 1.19 ± 0.02). These comparisons refer to types of engagement rather than use of specific social media platforms. The overall social media health information engagement index demonstrated a survey-weighted mean score of 1.55 ± 0.02, suggesting relatively modest engagement with health-related social media activities among U.S. adults.

### Reliability and dimensionality of the engagement index

The five social media engagement items demonstrated acceptable internal consistency, with a standardized Cronbach's alpha of 0.770. Item-rest correlations ranged from 0.323 for social media visitation to 0.668 for sharing general health-related information. In the one-factor exploratory factor analysis, loadings ranged from 0.359 for social media visitation to 0.773 for sharing general health-related information. These findings provided adequate support for retaining the overall engagement index as a secondary continuous outcome.

### Multivariable regression analyses

Table 2 presents survey-weighted multivariable analyses examining associations between political ideology, sociodemographic characteristics, and multiple dimensions of social media health-information engagement among U.S. adults. It mainly reports model-specific sample sizes, adjusted estimates, 95% confidence intervals, and joint Wald test *p*-values for political ideology. All six multivariable models were statistically significant overall based on design-adjusted *F* tests. For the five ordinal engagement outcomes, results are reported as adjusted odds ratios, while the overall engagement index is reported using unstandardized linear regression coefficients. The survey-weighted linear regression model for the overall engagement index was statistically significant, *F*_(21,176)_ = 28.45, *p* < 0.001, and explained approximately 14.4% of the variance in overall engagement (*R*^2^ = 0.144). Age was consistently associated with lower engagement across all outcomes. Each additional year of age was associated with lower odds of more frequent social media visitation (aOR = 0.950, 95% CI [0.944–0.956]), sharing personal health information (aOR = 0.983, 95% CI [0.975–0.991]), sharing general health information (aOR = 0.985, 95% CI [0.979–0.990]), interacting with others online (aOR = 0.977, 95% CI [0.970–0.983]), and watching health-related videos (aOR = 0.972, 95% CI [0.967–0.977]). Age was also negatively associated with the overall engagement index (*β* = −0.013, 95% CI [−0.015 to 0.011]).

**Table 2:** Survey-weighted associations between political ideology, sociodemographic characteristics, and social media health-information engagement among U.S. adults, HINTS 2024.

Variables	Visited social media (*N* = 5,850)	Shared personal content (*N* = 5,831)	Shared general content (*N* = 5,824)	Interacted with others (*N* = 5,843)	Watched videos (*N* = 5,850)	Overall engagement (*N* = 5,849)
Age	0.950 [0.944–0.956]***	0.983 [0.975–0.991]***	0.985 [0.979–0.990]***	0.977 [0.970–0.983]***	0.972 [0.967–0.977]***	−0.013 [−0.015 to 0.011]***
Female	1.673 [1.411–1.983]***	1.372 [1.091–1.726]**	1.608 [1.316–1.964]***	1.732 [1.354–2.216]***	1.272 [1.082–1.495]**	0.169 [0.107 to 0.231]***
Race and ethnicity						
Ref: NH White						
NH Black	0.925 [0.687–1.247]	0.966 [0.690–1.353]	0.787 [0.610–1.017]	0.967 [0.703–1.331]	1.359 [1.051–1.756]*	0.027 [−0.053 to 0.107]
Hispanic	0.913 [0.701–1.189]	1.127 [0.818–1.551]	0.890 [0.665–1.190]	0.868 [0.653–1.153]	1.550 [1.222–1.967]***	0.058 [−0.020 to 0.136]
NH Asian	0.816 [0.508–1.310]	1.161 [0.596–2.260]	1.473 [0.877–2.474]	1.207 [0.723–2.017]	2.043 [1.496–2.791]***	0.157 [−0.012 to 0.326]
NH Other	0.794 [0.489–1.290]	1.432 [0.832–2.467]	1.088 [0.668–1.772]	0.844 [0.478–1.490]	0.981 [0.677–1.422]	0.036 [−0.117 to 0.189]
Educational attainment						
Ref: Less than high school						
High school graduate	1.862 [1.294–2.679]***	0.978 [0.444–2.155]	1.104 [0.520–2.344]	0.763 [0.424–1.375]	0.976 [0.636–1.496]	0.031 [−0.164 to 0.226]
Some college	1.797 [1.198–2.697]**	1.098 [0.524–2.300]	1.398 [0.658–2.971]	1.204 [0.633–2.288]	1.304 [0.886–1.919]	0.130 [−0.068 to 0.329]
Bachelor's degree	1.488 [0.969–2.284]	1.284 [0.605–2.728]	1.367 [0.611–3.059]	1.157 [0.582–2.302]	1.336 [0.881–2.028]	0.116 [−0.096 to 0.328]
Post-baccalaureate	1.272 [0.838–1.930]	1.118 [0.494–2.530]	1.638 [0.696–3.856]	1.251 [0.624–2.505]	1.334 [0.864–2.058]	0.105 [−0.114 to 0.324]
Household income						
Ref: Less than $20,000						
$20,000–<$35,000	1.492 [1.017–2.188]*	0.656 [0.391–1.101]	1.010 [0.653–1.561]	0.867 [0.577–1.302]	1.221 [0.905–1.648]	0.040 [−0.099 to 0.178]
$35,000–<$50,000	1.772 [1.205–2.605]**	0.852 [0.552–1.315]	0.847 [0.612–1.173]	0.743 [0.539–1.023]	1.141 [0.849–1.533]	0.004 [−0.097 to 0.105]
$50,000–<$75,000	1.942 [1.333–2.829]***	0.600 [0.395–0.912]*	0.878 [0.604–1.275]	0.781 [0.532–1.148]	1.412 [1.011–1.972]*	0.032 [−0.081 to 0.146]
$75,000–<$100,000	2.160 [1.529–3.050]***	0.681 [0.438–1.060]	0.939 [0.663–1.330]	0.912 [0.623–1.335]	1.607 [1.114–2.317]*	0.082 [−0.024 to 0.188]
$100,000 or greater	2.093 [1.540–2.843]***	0.433 [0.289–0.647]***	0.561 [0.396–0.794]***	0.594 [0.412–0.856]**	1.196 [0.863–1.658]	−0.054 [−0.152 to 0.043]
Political ideology						
Ref: Moderate						
Very conservative	1.380 [0.990–1.926]	0.910 [0.549–1.510]	0.939 [0.614–1.435]	0.994 [0.644–1.534]	0.820 [0.547–1.229]	0.002 [−0.124 to 0.128]
Conservative	1.095 [0.815–1.471]	0.824 [0.539–1.259]	0.704 [0.500–0.990]*	0.932 [0.667–1.302]	0.863 [0.664–1.121]	−0.064 [−0.150 to 0.021]
Somewhat conservative	1.160 [0.875–1.538]	0.798 [0.497–1.283]	0.969 [0.732–1.283]	1.220 [0.867–1.718]	1.151 [0.934–1.420]	−0.027 [−0.108 to 0.053]
Somewhat liberal	1.060 [0.756–1.486]	0.709 [0.449–1.120]	0.836 [0.597–1.171]	1.178 [0.828–1.678]	1.116 [0.864–1.441]	−0.014 [−0.118 to 0.090]
Liberal	1.110 [0.836–1.474]	1.081 [0.738–1.581]	1.264 [0.931–1.716]	1.052 [0.711–1.556]	0.908 [0.723–1.140]	0.007 [−0.106 to 0.119]
Very liberal	1.387 [0.969–1.985]	1.415 [0.893–2.240]	1.365 [0.945–1.970]	2.144 [1.452–3.166]***	1.017 [0.682–1.517]	0.131 [0.001 to 0.261]*
Overall model *F*	18.17***	4.56***	6.96***	8.05***	14.23***	28.45***
Joint Wald test for political ideology, *p*-value	0.436	0.104	0.014	0.003	0.320	0.121
*R* ^2^	NA	NA	NA	NA	NA	0.144

Estimates for the five individual engagement outcomes are adjusted odds ratios with 95% confidence intervals in square brackets from survey-weighted ordinal logistic regression models. Estimates for overall engagement are unstandardized coefficients with 95% confidence intervals in square brackets from survey-weighted linear regression. Reference categories were male, non-Hispanic White, less than high school education, household income below $20,000, and moderate political ideology. Overall model *F* statistics are design-adjusted. *R*^2^ is reported only for the linear regression model because conventional *R*^2^ is not directly applicable to ordinal logistic regression. All models incorporated HINTS sampling weights, strata, and clustering variables. Ref = reference category. Model-specific sample sizes differ because of outcome-specific missingness and complete-case inclusion of political ideology and sociodemographic covariates. No missing values were imputed.

* *p* < 0.05.

** *p* < 0.01.

*** *p* < 0.001.

Female participants had higher odds of more frequent engagement than male participants across all five individual outcomes, including social media visitation (aOR = 1.673, 95% CI [1.411–1.983]), sharing personal health information (aOR = 1.372, 95% CI [1.091–1.726]), sharing general health information (aOR = 1.608, 95% CI [1.316–1.964]), interacting with others online (aOR = 1.732, 95% CI [1.354–2.216]), and watching health-related videos (aOR = 1.272, 95% CI [1.082–1.495]). Female participants also had higher overall engagement scores (*β* = 0.169, 95% CI [0.107–0.231]). Compared with non-Hispanic White participants, non-Hispanic Black, Hispanic, and non-Hispanic Asian participants had higher odds of more frequent video-based engagement. The associations were strongest among non-Hispanic Asian participants (aOR = 2.043, 95% CI [1.496–2.791]) and Hispanic participants (aOR = 1.550, 95% CI [1.222–1.967]). Educational attainment was primarily associated with social media visitation, while household income showed differing associations across engagement behaviors. Compared with participants reporting household incomes below $20,000, those in higher income categories generally had greater odds of more frequent social media visitation. However, participants with household incomes of $100,000 or greater had lower odds of sharing personal health information (aOR = 0.433, 95% CI [0.289–0.647]), sharing general health information (aOR = 0.561, 95% CI [0.396–0.794]), and interacting with others online (aOR = 0.594, 95% CI [0.412–0.856]).

Political ideology was jointly associated with sharing general health information [*F*_(6,191)_ = 2.75, *p* = 0.014] and interacting online with individuals who had similar health or medical concerns [*F*_(6,191)_ = 3.46, *p* = 0.003). Compared with politically moderate participants, conservative participants had lower odds of more frequent sharing of general health information (aOR = 0.704, 95% CI [0.500–0.990]), while very liberal participants had higher odds of more frequent online interaction (aOR = 2.144, 95% CI [1.452–3.166]). Very liberal participants also had a slightly higher overall engagement score than moderate participants (*β* = 0.131, 95% CI [0.001–0.261]); however, the joint test for political ideology was not statistically significant for the overall engagement index (*p* = 0.121). Political ideology was also not jointly associated with social media visitation, sharing personal health information, or watching health-related videos. Overall, sociodemographic characteristics, particularly age and sex, showed more consistent associations with engagement than political ideology.

## Discussion

This study examined associations between political ideology, sociodemographic characteristics, and multiple dimensions of social media health-information engagement among U.S. adults using nationally representative HINTS 2024 data. Political ideology was associated with selected forms of engagement, although the pattern was not consistent across all outcomes. Ideology was jointly associated with sharing general health information and interacting online with individuals who had similar health or medical concerns. Compared with politically moderate participants, conservative participants had lower odds of more frequent general health-information sharing, whereas very liberal participants had higher odds of more frequent online interaction. In contrast, ideology was not jointly associated with social media visitation, sharing personal health information, watching health-related videos, or the overall engagement index. These findings suggest that ideological differences may be more relevant to active information exchange and interpersonal interaction than to general social media use. This interpretation is consistent with research showing that social media can facilitate content sharing, selective exposure, ideologically aligned interaction, and reciprocal information flows among citizens, political actors, and news organizations (41, 42, 48, 49). However, the observed associations may also reflect differences in institutional trust, communication preferences, social networks, or the types of health information encountered online. These findings should not be interpreted as evidence that political polarization occurred. HINTS included a single measure of ideological self-placement but did not assess movement toward ideological extremes, policy-specific divergence, hostility toward opposing political groups, or perceived ideological distance. Consequently, the results represent differences in selected health-information engagement behaviors across self-identified ideological groups rather than evidence of ideological, affective, or false polarization. The cross-sectional design also does not establish whether political ideology influenced engagement, whether engagement contributed to ideological differences, or whether both were associated with other unmeasured factors. Although political ideology was a central exposure in this study, age, sex, education, income, and race and ethnicity demonstrated more consistent associations across the engagement outcomes and therefore provide important complementary evidence regarding variation in social media health-information engagement among U.S. adults.

First, age was consistently associated with lower levels of social media health information engagement across all engagement dimensions. Second, higher educational attainment was associated with greater engagement, particularly for active digital behaviors such as content sharing and online interaction. Third, important racial and ethnic differences emerged across several forms of social media engagement. Finally, political ideology demonstrated smaller but meaningful associations with selected digital engagement behaviors. The strong negative association between age and social media engagement is consistent with prior research demonstrating persistent digital divides among older adults (50, 51). Older individuals may experience lower digital literacy, reduced familiarity with social media platforms, and fewer opportunities for digital engagement compared with younger populations (52, 53). These disparities may have important implications for access to online health information, participation in digital health communication environments, and exposure to public health messaging delivered through social media platforms. As healthcare systems and public health organizations increasingly rely on digital communication strategies, lower engagement among older adults may contribute to widening disparities in digital health access and information utilization. The strong associations observed for age and sex are consistent with previous HINTS-based research. Prior national analyses found that younger adults and women were more likely to engage in multiple social media health activities, suggesting that these patterns have persisted across survey cycles. The present findings extend that literature by examining five distinct engagement frequencies rather than treating social media health use as a single binary behavior.

Educational attainment emerged as one of the strongest predictors of social media health information engagement. Individuals with higher education levels demonstrated consistently greater engagement across multiple digital behaviors, suggesting that educational inequalities may contribute to disparities in digital health communication and access to online health information resources (13, 54). Higher educational attainment may be associated with greater digital literacy, stronger information-seeking skills, and greater familiarity navigating online information environments. Similarly, higher household income was associated with several dimensions of engagement, further highlighting the importance of socioeconomic factors in shaping digital health participation and online health communication behaviors (55).

Several racial and ethnic differences in social media health information engagement were also observed. Hispanic participants demonstrated significantly greater video-based engagement and higher overall social media engagement, while non-Hispanic Asian participants reported greater engagement across several digital behaviors, including content sharing and video consumption. These findings may reflect differences in communication preferences, cultural patterns of social media use, health information-seeking behaviors, and reliance on digital platforms for accessing health-related information. The growing use of video-based health communication among minority populations may present important opportunities for culturally tailored public health messaging and digital health interventions.

Several important public health implications emerge from these findings. Public health organizations and healthcare institutions should prioritize accessible, evidence-based, and culturally inclusive digital communication strategies to improve engagement with online health information across diverse populations. Efforts to improve digital health literacy may be particularly important for older adults and individuals with lower educational attainment, who have consistently demonstrated lower levels of engagement. In addition, understanding differences in digital engagement behaviors across political and sociodemographic groups may help public health organizations develop more effective targeted communication strategies for disseminating health information through social media platforms. These findings also have implications for misinformation-sensitive health communication. Public health organizations should consider that engagement frequency does not necessarily indicate exposure to accurate or evidence-based information. Communication strategies should therefore address both access and information quality by using credible messengers, transparent sourcing, accessible language, and formats appropriate for different types of social media engagement. Because perceived health misinformation may be associated with lower institutional trust, communication approaches should avoid assuming that a single message or platform will be equally effective across ideological groups.

This study has several limitations. First, the cross-sectional design prevents causal inference regarding relationships between political ideology, sociodemographic characteristics, and social media engagement behaviors. Second, the political ideology measure captured self-placement on the liberal-conservative continuum but did not directly assess political polarization. Longitudinal data or measures of ideological distance would be needed to evaluate ideological polarization; measures of out-group dislike or distrust would be required to assess affective polarization; and perceived and actual group-position measures would be needed to evaluate false polarization. Third, HINTS did not identify the specific platforms on which respondents engaged with health information. The study therefore cannot determine whether the observed activities occurred primarily on Facebook, YouTube, Instagram, TikTok, X, or another platform, nor can it compare or rank platform use. Platform-specific research is needed because platform design, user populations, recommendation algorithms, and content formats may influence health-information engagement differently. Finally, social media engagement behaviors may be influenced by additional psychosocial, cultural, or technological factors that were not fully captured in the current analyses. Despite these limitations, this study provides important nationally representative evidence regarding political ideology, socioeconomic disparities, and multidimensional social media health information engagement among U.S. adults.

## Conclusion

Using nationally representative HINTS 2024 data, this study found that sociodemographic characteristics and political ideology were associated with multiple dimensions of social media health information engagement among U.S. adults. Age was consistently associated with lower engagement across all social media behaviors, while higher educational attainment was associated with greater digital engagement. Several racial and ethnic differences also emerged, particularly for video-based engagement and overall social media use. Political ideology demonstrated smaller but meaningful associations with selected engagement behaviors, suggesting that political ideology was associated with selected engagement behaviors, although these associations were less consistent than those observed for age and sex. These findings highlight important digital public health disparities in social media health information engagement and emphasize the need for inclusive, accessible, and targeted digital health communication strategies.

## Data Availability

Publicly available datasets were analyzed in this study. This data can be found here: https://hints.cancer.gov/.
